# In a randomized trial in prostate cancer patients, dietary protein restriction modifies markers of leptin and insulin signaling in plasma extracellular vesicles

**DOI:** 10.1111/acel.12657

**Published:** 2017-09-17

**Authors:** Erez Eitan, Valeria Tosti, Caitlin N. Suire, Edda Cava, Sean Berkowitz, Beatrice Bertozzi, Sophia M. Raefsky, Nicola Veronese, Ryan Spangler, Francesco Spelta, Maja Mustapic, Dimitrios Kapogiannis, Mark P. Mattson, Luigi Fontana

**Affiliations:** ^1^ Laboratory of Neurosciences National Institute on Aging, NIH 251 Bayview Boulevard Baltimore MD 21224 USA; ^2^ Division of Geriatrics and Nutritional Sciences and Center for Human Nutrition Washington University School of Medicine St. Louis MO 63110 USA; ^3^ Department of Medicine (DIMED) Geriatrics Division University of Padova 35128 Padova Italy; ^4^ Department of Medicine University of Verona 37129 Verona Italy; ^5^ Department of Neuroscience Johns Hopkins University School of Medicine 725 N. Wolfe Street Baltimore MD 21205 USA; ^6^ Department of Clinical and Experimental Sciences Brescia University 25121 Brescia Italy; ^7^ CEINGE Biotecnologie Avanzate 80122 Napoli Italy

**Keywords:** exosomes, extracellular vesicles, IRS‐1, leptin receptor, prostate cancer, protein restriction

## Abstract

Obesity, metabolic syndrome, and hyperleptinemia are associated with aging and age‐associated diseases including prostate cancer. One experimental approach to inhibit tumor growth is to reduce dietary protein intake and hence levels of circulating amino acids. Dietary protein restriction (PR) increases insulin sensitivity and suppresses prostate cancer cell tumor growth in animal models, providing a rationale for clinical trials. We sought to demonstrate that biomarkers derived from plasma extracellular vesicles (EVs) reflect systemic leptin and insulin signaling and respond to dietary interventions. We studied plasma samples from men with prostate cancer awaiting prostatectomy who participated in a randomized trial of one month of PR or control diet. We found increased levels of leptin receptor in the PR group in total plasma EVs and in a subpopulation of plasma EVs expressing the neuronal marker L1CAM. Protein restriction also shifted the phosphorylation status of the insulin receptor signal transducer protein IRS1 in L1CAM+ EVs in a manner suggestive of improved insulin sensitivity. Dietary PR modifies indicators of leptin and insulin signaling in circulating EVs. These findings are consistent with improved insulin and leptin sensitivity in response to PR and open a new window for following physiologic responses to dietary interventions in humans.

Obesity and insulin resistance are associated with accelerated aging and increased risk of many age‐related diseases (Mattson *et al*., 2016). The risk of many cancers, including prostate cancer, increases with age and being overweight further increases this risk (Longo & Fontana, 2010; Neuhouser *et al*., 2015). A protein restriction (PR) diet was shown to reduce weight and improve metabolic health in both animal models and humans, and researchers have started to elucidate beneficial effects of dietary PR in cancer patients (Fontana *et al*., 2013, 2016; Kopeina *et al*., 2017). The mechanism by which PR diet improves metabolism and slows down cancer progression is not yet fully understood. One reasonable hypothesis is that PR modulates the levels of adipokines and other endocrine signals that affect cancer progression. For instance, elevated blood concentration of FGF21 and adiponectin and reduced levels of leptin were previously observed in humans and animal models in response to PR diet (Johnson *et al*., 2007; Harvie *et al*., 2013; Fontana *et al*., 2016).

In the current study, we are investigating the effect of PR diet on molecular mediators in Extracellular vesicles (EV). EVs are small membranous particles that serve in intercellular communication by delivering proteins, RNA, DNA, and bioactive lipids (Yanez‐Mo *et al*., 2015). We postulate that EV‐bound molecules reflect cellular and metabolic processes in their cells of origin, which may be modulated by PR diet and serve as biomarkers for following personalized diet responses. Here, we used a two‐step method (first precipitating total EVs, then, immunoprecipitating EVs that express L1CAM to generate a subpopulation enriched for neuronal origin) (Kapogiannis *et al*., 2015; Mustapic *et al*., 2017) to isolate EVs from plasma samples (1 ml) of 38 subjects at baseline and following 1 month of dieting. Subjects were men with prostate cancer awaiting prostatectomy surgery, most of whom were overweight (BMI = 30.45 ± 5.8; Age = 59.26 ± 7.5 years), that were randomly assigned to either a control diet or a PR diet. The PR diet was individualized so that each patient received 0.8 g protein kg^−1^ lean body mass; consequently, PR meals were provided by the investigators, while the control group maintained their regular diet. This difference in handling subjects was dictated by practical considerations and might be considered as a confounding factor. For further description of the study design, macronutrient breakdown and subjects baseline characteristics see (Fontana *et al*., 2016), (Fig. S1; Table S1; Appendix S1). The quality of the EV preparations was evaluated by nanoparticle tracking analysis and Western blots for EV markers ALIX and CD9 (Fig. 1). The isolated EVs had a typical diameter size distribution, with the majority having diameters between 100 and 180 nm (Fig. 1A). The EV preparations showed expression of canonical EV markers ALIX and CD9 (Fig. 1B). There were no significant changes in EV size distributions from baseline to after 1 month of either diet (interaction of diet type and time point, F(1, 36) = 0.101, *P *=* *0.753) (Fig. 1A). EV concentration was included as a covariate in all subsequent analyses to control for differential EV content between samples.

**Figure 1:**
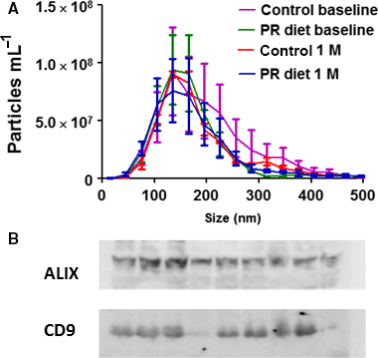
Characterization of EV populations and results of ELISA analysis of a select group of energy metabolism‐related proteins in L1CAM+ EVs. (A) The size distribution of L1CAM EVs from 19 control and 19 PR diet subjects at baseline and after 1 month of dietary intervention was measured by NTA. (B) Immunoblot analysis for the EV markers ALIX and CD9 in nine randomly chosen samples of isolated L1CAM EVs.

After establishing that EV concentration is not changing in response to the PR diet, we shifted our attention to their content, focusing on leptin receptor (LeR) and the insulin signaling protein IRS1. While levels of leptin and insulin have been extensively measured in plasma samples of obese and aged individuals (Gumbiner *et al*., 1989; Balasko *et al*., 2014), their receptors and downstream signaling mediators are not being routinely measured in human studies. The isolation of EVs opens the opportunity to measure these nonhormonal protein mediators and hopefully assess cellular responses to the diets in living humans. Moreover, while measuring levels of soluble factors in plasma only gives indications of systemic changes, plasma EVs can be sorted based on their surface proteins to study cell‐origin specific populations. We published several studies on plasma‐derived neuronal‐enriched EVs and their potential to open a window into phenotypic changes of the human brain (Kapogiannis *et al*., 2015; Mustapic *et al*., 2017). The plasma‐derived neuronal‐enriched EVs are selected based on the presence of the neuronal marker L1CAM on their surface. We previously demonstrated that they represent a distinct subpopulation that is enriched for several neuronal proteins and interestingly also contain elevated levels of hormone receptors and intracellular signaling molecules like mTOR, IRS1, and leptinR (Mustapic *et al*., 2017) compared to total EVs, perhaps, a reflection of the high neuronal metabolic activity. Leptin is released from adipocytes and activates receptors on neurons in the brain that enhance satiety, thereby inhibiting excessive food intake (Park & Ahima, 2015). Obese humans typically experience a heightened sense of hunger despite elevated leptin levels, perhaps due to the development of leptin resistance (Gautron & Elmquist, 2011). The concentration of leptin in blood is elevated in obese individuals and is inversely correlated with age (Balasko *et al*., 2014). Here, we measured the levels of EV‐associated LeR and showed that the PR diet produced a significant increase from baseline and in comparison with the control diet (interaction of diet type and time point, F(1, 36) = 6.52, *P *=* *0.015; univariate post hoc comparison of the two diets after 1 month, F(1, 43) = 5.579, *P *=* *0.021; univariate post hoc comparison between baseline and 1 month after PR diet, F(1, 36) = 9.311, *P *=* *0.004) (Fig. 2A). The PR diet produced an even greater and highly significant LeR increase from baseline and compared to the control diet in neuronal‐enriched EVs (interaction of diet type and time point, F(1, 36) = 20.120, *P *<* *0.001; univariate post hoc comparison of the two diets after 1 month, F(1, 43) = 6.41, *P *=* *0.015; univariate post hoc comparison between baseline and 1 month after PR diet, F(1, 35) = 52.772, *P *<* *0.001) (Fig. 2B). In other words, 1 month of PR increased LeR levels in total and neuronal‐enriched EVs, unlike the control diet.

**Figure 2:**
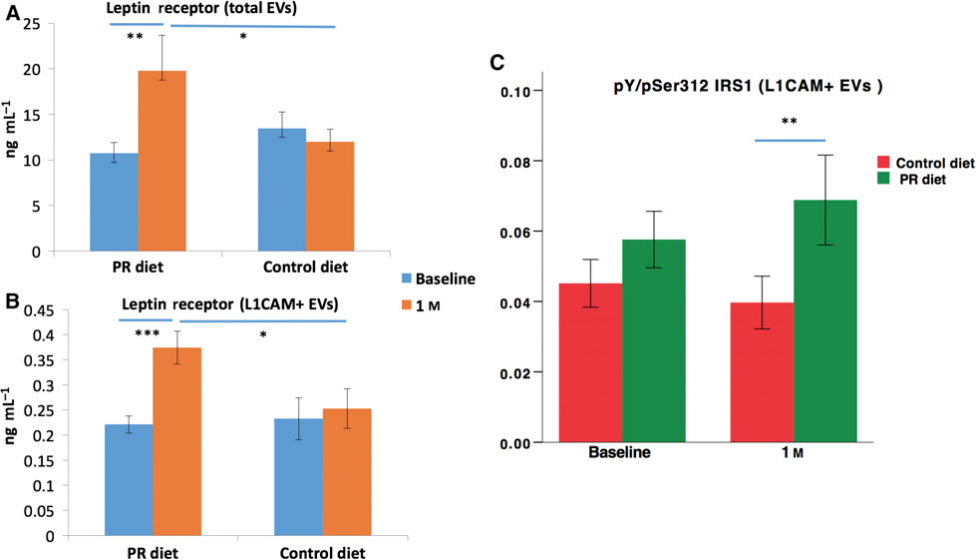
Levels of leptin receptor and the pY/pSer312 IRS1 ratio in EVs. (A) Levels of leptin receptor after a month of PR diet were significantly higher than baseline levels and control levels in total EVs. (B) Levels of leptin receptor after a month of PR diet were significantly higher than baseline levels and control levels in L1CAM+ EVs. (C) The ratio between pan‐tyrosine and serine 312 phosphorylated IRS1 in L1CAM+ EVs. Bars depict means, and error bars depict SEM; * indicates significance < 0.05; ** indicates significance < 0.01; and *** indicates significance < 0.001.

Insulin is an important regulator of metabolism, and proper insulin signaling is critical for cellular health. Reduction in insulin sensitivity is the underlying cause of type 2 diabetes and a characteristic of obesity and unhealthy aging (Bartke, 2008). IRS1 is an adapter protein that mediates the downstream signaling of insulin and insulin‐like growth factor receptors. Phosphorylation of IRS1 on tyrosine residues increases its activity while phosphorylation on serine 312 decreases its activity (Copps & White, 2012). The ratio of pan‐tyrosine (pY) to serine 312 phosphorylated (pSer312) IRS1 (Y/S IRS1 ratio) was shown to decrease in response to insulin resistance in cell studies (Copps & White, 2012), and we have recently shown that it is significantly decreased in neuronal‐enriched EVs of patients with Alzheimer's disease (AD) compared to controls (Kapogiannis *et al*., 2015), in whom it is also associated with regional atrophy (Mullins *et al*., 2017). The PR diet was previously reported to increase insulin sensitivity in these subjects (measured by HOMA‐IR) (Fontana *et al*., 2016), and therefore, we were interested to test whether it also changes the Y/S IRS1 ratio in neuronal‐enriched EVs, which would further validate its use as a biomarker reflecting neuronal insulin sensitivity. After 1 month of PR diet, subjects had higher Y/S IRS1 ratio in neuronal‐enriched EVs compared to subjects on control diet (interaction of diet type and time point, F(1, 35) = 1.259, *P *=* *0.269; univariate post hoc comparison between PR diet and control diet after 1 month, F(1, 67) = 8.889, *P *=* *0.004). This difference indicates a more active insulin signaling pathway after PR diet compared to control diet (Fig. 2C). To assess whether baseline BMI affects LeR and Y/S IRS1 ratio results, we explored models, in which baseline BMI was included as covariate, as well as the triple interaction term ‘timepoint*treatment*BMIbaseline’. Neither BMI nor the interaction term reached significance; therefore, we report simpler models without covarying baseline BMI.

The results of the current study further show that circulating EVs can serve as a source of biomarkers for cellular metabolism. PR diet, which was previously reported to significantly reduce BMI, increase insulin sensitivity and FGF21 concentration and produce a trend toward reduced prostate‐specific antigen (PSA) levels in urine (Fontana *et al*., 2016), also increased the levels of EV‐associated LeR and Y/S IRS1 ratio. There are two major advantages of EV‐based biomarkers. First, EV content is directly derived from the intracellular compartment and their levels of cellular mediators like IRS1 and LeR may better represent cellular metabolic status than circulating soluble mediators. In this regard, it will be interesting to measure additional metabolism‐related proteins, such as mTOR, which we have recently shown to be present in EVs (Mustapic *et al*., 2017), in its native and phosphorylated active forms, as it is known to regulate IRS1 phosphorylation (Chiarini *et al*., 2015). Second, EVs express cell specific proteins on their surface (Yanez‐Mo *et al*., 2015), which can be used as a handle to enrich for EVs originating from specific cell types (Mustapic *et al*., 2017), potentially allowing conclusions to be drawn about diet effects on specific cell types. Further animal studies are needed to establish the association between the levels of these biomarkers in circulating EVs and in their cells of origin to determine whether they provide an accurate insight into the molecular alterations occurring in different tissues. In particular, insulin resistance of specific cell types is significant for several age‐related diseases. In this regard, we have found reduced Y/S IRS1 ratio in AD patients, which likely corresponds to increased insulin resistance, particularly affecting brain regions suffering from AD pathology (Talbot *et al*., 2012; Kapogiannis *et al*., 2015; Mullins *et al*., 2017). Our present results show that PR diet increases the Y/S IRS1 ratio in neuronal‐enriched EVs, which suggests that this diet may improve insulin sensitivity in neurons. These results suggest an explanation for the vast body of findings in animal studies indicating that PR and calorie restriction can counteract several major age‐related diseases (Mattson *et al*., 2016; Simpson *et al*., 2017). The reduction in sensitivity to insulin, leptin, and probably other hormones and trophic factors with age is probably one of the drivers of aging and an underlying cause of age‐related diseases. The ability to monitor this process in humans longitudinally by a minimally invasive intervention such as a blood draw is of great value for understanding mechanisms of aging, for monitoring age‐related disease processes and for assessing responses of systemic and tissue‐specific metabolism to therapeutic interventions including diet, exercise, lifestyle modifications and drugs (Longo *et al*., 2015). We believe that EVs offer a unique ‘window’ into these processes and that the current study provides a proof of concept for their use as a source of biomarkers for age‐related metabolism alterations.

## **Funding**

No funding information provided.

## Conflict of interest

The authors declare that they have no conflict of interest.

## **Author contributions**

L. Fontana, D. Kapogiannis, and M. Mattson had the original idea; L. Fontana wrote the clinical study protocol; D. Kapogiannis, E. Eitan, M. Mattson, and L. Fontana designed the EV study based on samples and data from the clinical study; N. Veronese, F. Spelta, E. Cava, and V. Tosti identified, treated, and monitored study participants and contributed to data recording and analyses; B. Bertozzi prescribed PR or control diet and monitored compliance to the recommended diets; E. Eitan, C. Suire, S. Berkowitz, S. Raefsky, R. Spangler, and Maja Mustapic performed EV isolation and biomarker measurements; D. Kapogiannis performed the statistical analyses; E. Eitan, D. Kapogiannis, M. Mattson, and L. Fontana contributed to data analyses and interpretation; E. Eitan, D. Kapogiannis, M. Mattson, and L. Fontana wrote the final version of the manuscript. All the authors had direct access to original data, critically revised the draft, and approved the final manuscript. D. Kapogiannis and L. Fontana are the guarantors and take final responsibility for the contents of the manuscript. No medical writer was involved.

## Supporting information


**Fig. S1** Protein Restriction and Prostate Cancer Study Flow Diagram.
**Table S1** Body composition, plasma lipids and PSA levels at baseline and after 1 month of dietary intervention.

**Appenix S1.** Subjects, experimental procedures and methods.
