# Supplementary material for: In a randomized trial in prostate cancer patients, dietary protein restriction modifies markers of leptin and insulin signaling in plasma extracellular vesicles
Source: Aging Cell. 2017 Sep 17;16(6):1430–3. doi: 10.1111/acel.12657 (PMC5676054; doi:10.1111/acel.12657)
Supplement: Supplementary file 1 — Fig. S1 Protein Restriction and Prostate Cancer Study Flow Diagram. Table S1 Body composition, plasma lipids and PSA levels at baseline and after 1 month of dietary intervention. [file ACEL-16-1430-s001.docx]

|  | **PR**  **(n=19)** | **Controls**  **(n=19)** | | **Between-group *p**** |
| --- | --- | --- | --- | --- |
| **Age,** ys | 58.74±7.5 (1.72) | 59.79±6.80 (1.56) | | 0.65 |
| **Ht**, cm | 181.86±9.48 (2.17) | 175.71±6.71 (1.54) | | 0.03 |
| **Wt,** Kg |  |  | |  |
| BL | 101.46±18.85 (4.32) | 93.17±17.25 (3.96) | | 0.17 |
| 1month-FU | 98.85±18.61 (4.27) | 92.96±16.87 (3.87) | | 0.31 |
| Change | -2.62±2.17 (0.50) | -0.21±1.36 (0.31) | | **<0.0001^na^** |
| Within-group P | <0.0001 | 0.52 | | **0.001** |
| **BMI,** Kg/m^2^ |  |  | |  |
| BL | 30.67±5.42 (1.24) | 30.24±5.79 (1.33) | | 0.82 |
| 1month-FU | 29.88±5.32 (1.22) | 30.18±5.67 (1.30) | | 0.87 |
| Change | -0.79±0.69 (0.16) | -0.07±0.43 (0.99) | | **<0.0001** |
| Within-group P | <0.0001 | 0.51 | | **<0.0001** |
| **TRUNK tissue % fat** |  |  | |  |
| BL | 38.55±9.44 (2.16) | 38.36±9.86 (2.26) | | 0.95 |
| 1month-FU | 37.87±9.26 (2.12) | 38.15±9.95 (2.28) | | 0.93 |
| Change | -0.67±1.35 (0.31) | -0.21±1.33 (0.31) | | **0.30** |
| Within-group P | 0.04 | 0.49 | | **0.31** |
| **TRUNK fat, g** |  |  | |  |
| BL | 19835.26±7756.38 (1179.43) | 18771.84±8857.06 (2031.95) | | 0.70 |
| 1month-FU | 18787.26±7193.11 (1650.21) | 18652.58±8686.59 (1992.84) | | 0.96 |
| Change | -1048.00±1109.78 (254.60) | -119.26 ±778.59 (178.62) | | **0.005** |
| Within-group P | 0.001 | 0.51 | | **0.004** |
| **TOT tissue % fat** |  | |  |  |
| BL | 33.51±6.94 (1.59) | | 33.87±7.43 (1.70) | 0.88 |
| 1month-FU | 33.11±6.70 (1.54) | | 33.62±7.33 (1.68) | 0.83 |
| Change | -0.39±0.89 (0.20) | | -0.25±0.96 (0.22) | **0.63** |
| Within-group P | 0.07 | | 0.28 | **0.60** |
| **TOT fat, g** |  | |  |  |
| BL | 33237.05±11908.33 (2731.96) | | 31073.53±11834.18 (2714.95) | 0.58 |
| 1month-FU | 31850.68±11092.85 (2544.87) | | 30716.10±11450.60 (2626.95) | 0.76 |
| Change | -1386.37±1551.88 (356.02) | | -357.42±1024.41 (235.01) | **0.02** |
| Within-group P | 0.001 | | 0.15 | **0.02** |
| **TOT lean mass, g** |  |  | |  |
| BL | 63053.63±8180.06 (1876.63) | 57983.16±7122.68 (1634.05) | | 0.05 |
| 1month-FU | 61632.47±8125.29 (1864.07) | 57961.05±6853.20 (1572.23) | | 0.14 |
| Change | -1421.16±1266.60 (290.58) | -22.10±1182.26 (271.23) | | **0.001** |
| Within-group P | <0.0001 | 0.94 | | **0.005** |
| **TChol,** mg/dl |  |  | |  |
| BL | 169.10±32.10 (7.36) | 182.79±33.31 (7.64) | | 0.21 |
| 1month-FU | 159.21±32.55 (7.47) | 185.63±33.71 (7.73) | | 0.02 |
| Change | -9.89±23.69 (5.43) | 2.84±15.89 (3.65) | | **0.06** |
| Within-group P | 0.09 | 0.45 | | **0.03** |
| **TG,** mg/dl |  |  | |  |
| BL | 90.68±51.44 (11.80) | 126.79±101.95 (23.39) | | 0.18 |
| 1month-FU | 110.58±37.53 (8.61) | 150.10±93.75 (21.51) | | 0.10 |
| Change | 19.89±40.09 (9.20) | 23.31±59.38 (13.62) | | **0.84** |
| Within-group P | 0.04 | 0.10 | | **0.33** |
| **HDL,** mg/dl |  |  | |  |
| BL | 49.53±14.59 (3.35) | 49.31±15.74 (3.61) | | 0.97 |
| 1month-FU | 48.16±14.48 (3.32) | 48.16±17.93 (4.11) | | 1.00 |
| Change | -1.37±4.77 (1.09) | -1.16±5.34 (1.22) | | **0.90** |
| Within-group P | 0.23 | 0.36 | | **0.90** |
| **LDL,** mg/dl |  |  | |  |
| BL | 100.31±30.54 (7.01) | 108.47±29.97 (6.87) | | 0.41 |
| 1month-FU | 89.05±29.47 (6.76) | 107.21±30.00 (6.88) | | 0.07 |
| Change | -11.26±19.14 (4.39) | -1.26±13.53 (3.10) | | **0.07** |
| Within-group P | 0.02 | 0.69 | | **0.04** |
| **PSA,** ng/ml |  |  | |  |
| BL | 6.48±4.57 | 4.96±2.07 | | 0.19 |
| 1month-FU | 5.83±3.14 | 4.66±2.21 | | 0.20 |
| Change | -0.66±1.81 | -0.30±0.71 | | **0.41** |
| Within-group P | 0.09 | 0.13 | | **0.85** |

**Supplemental Table 1.** Body composition, plasma lipids and PSA levels at baseline and after 1 month of dietary intervention. The Table depicts mean values +/-SD (SE) per group at baseline (BL), at the 1 month follow-up visit (1month-FU), the absolute mean change (Change), the within-group level of significance for the change (Within-group P), and the between-groups level of significance for the various comparisons (Between-group p*). The first Between-group p* in bold is non-adjusted for the baseline value and the second Between-group p* in bold is adjusted for the baseline value.

**Supplemental Figure. Protein Restriction and Prostate Cancer Study Flow Diagram**

Control Diet

Protein Restriction Diet

Randomized (n=39)

Excluded (n=3032)

♦  Not meeting inclusion criteria (n=2076)

♦  Declined to participate (n=302)

♦  Other reasons (n=654)

Follow-Up

Analysed (n=19)
♦ Excluded from analysis (n=0)

Analysis

Analysed (n=19)
♦ Excluded from analysis (n=0)

Lost to follow-up (give reasons) (n=0)

Discontinued intervention (give reasons) (n=0)

Lost to follow-up (give reasons) (n=0)

Discontinued intervention (n=0)

Enrollment

Allocated to intervention (n=19)

**♦ Received allocated intervention (n=19)**

♦ Did not receive allocated interventio (n=0)

Allocation

Assessed for eligibility (n=3071) )
