# Supplementary material for: In a randomized trial in prostate cancer patients, dietary protein restriction modifies markers of leptin and insulin signaling in plasma extracellular vesicles
Source: Aging Cell. 2017 Sep 17;16(6):1430–3. doi: 10.1111/acel.12657 (PMC5676054; doi:10.1111/acel.12657)
Supplement: Supplementary file 2 — Appenix S1. Subjects, experimental procedures and methods. [file ACEL-16-1430-s002.docx]

**Subjects, Experimental Procedures and Methods**

**Subjects:** The Experimental design and report were prepared following the CONSORT standards for randomized clinical trials (clinicaltrials.gov identifier NCT01692587). The present study reports on secondary outcomes of the trial concerning EV biomarkers; the primary outcome (expression of genes and proteins of the PI-3K/Akt/mTOR pathway) will be reported separately. Volunteers were recruited under a protocol approved by the IRB (No. 201011804) of the Washington University in St. Louis based on established inclusion (untreated patients with a diagnosis of localized prostate cancer confirmed by pathology, otherwise healthy) and exclusion (any major medical condition and chronic diseases (except for untreated localized prostate cancer), cognitive impairment, therapy with corticosteroids or anabolic agents such as androgen, androgen precursor, rhGH in the 6 months prior to enrollment) criteria. All participants provided written informed consent prior to inclusion in the study. The procedures followed were in accordance with the ethical standards of the responsible institutional or regional committee on human experimentation or in accordance with the Helsinki Declaration of 1975 as revised in 1983. After baseline screening, volunteers were allocated with a 1:1 ratio into a control (n=19) or experimental diet group (PR, n=19). The control subjects continued eating as usual, whereas volunteers randomized to the isocaloric PR diet consumed the provided isocaloric PR experimental meals prepared by the Metabolic kitchen of Washington University. There have been no important changes to methods or outcomes after trial commencement. A study flow diagram is included.

Anthropometrics, body composition, and metabolic parameters concentrations in plasma samples collected following an overnight fast were measured at baseline and after 43±11 days on diet. Body weight was measured after an overnight fast, with the participant wearing a hospital gown. Height was measured without shoes to the nearest 0.1 cm. BMI was calculated as weight divided by the square of height (kg/m2). Whole-body and trunk fat mass, and lean mass were assessed by dual-energy x-ray absorptiometry (DXA, Delphi W, Hologic Corporation, Waltham, MA, software version 11.2). Intention-to-treat analyses were performed using SPSS software (SPSS Inc., Chicago, Illinois). Changes between and within groups were tested with analysis of covariance and paired t-tests. Statistical tests were two-tailed, with significance accepted at P<0.05. Results from these measures are reported on Table 1.

**PR Diet -** Each participant randomized to the PR diet was fed costumized isocaloric PR diets prepared by the Metabolic kitchen of the Washington University CARS. L. Fontana was responsible for the randomization. In the PR menus dietary protein intake was decreased to the Dietary Reference Intake (DRI) for total protein. For adult males the DRI is 56 grams per day. To provide the participants with isocaloric menus three calorie levels were available: 2300 calories, 2600 calories and 2900 calories. The menus were planned to provide a similar percent of calories from protein (8% to 9%) for the three calorie levels. The intakes of protein were 56 grams to 60 grams per day. Individualized energy intakes were estimated from a 4-day food diary and two formulas for estimating energy expenditure: Harris-Benedict equation and Cunningham equation (include body composition and physical activity factors). The PR participants received a calorie level that was estimated to maintain body weight. Compliance with the isocaloric lower protein diet for volunteers randomized to PR was assessed by means of the daily meal checklist.

**Samples and EV isolation:** A total of 76 plasma samples (0.5 ml each) drawn from 38 subjects at two time points were studied. The following protocol was used for total EV isolation followed by L1CAM enrichment from a starting volume of 0.5 ml plasma, as previously published (Goetzl *et al.* 2015; Kapogiannis *et al.* 2015; Mustapic *et al.* 2017). First, 200 µl of thromboplastin (Pacific Hemostasis Thromboplastin-D, cat. #100356 Thermo Fisher Scientific Inc,) was added to plasma samples, tubes were inverted and allowed to incubate at room temperature for 30 minutes. At the end of the incubation, 300 µl Dulbecco balanced salt solution with 3X protease (Complete Ultra tablet, mini Easy pack cat. #78420, Thermo Fisher Scientific Inc) and phosphatase (Halt phosphatase inhibitor, Pierce Halt, Thermo Scientific, cat#1862495) inhibitors was added , tubes were inverted, and samples were centrifuged at 3000 x g for 20 minutes at 4°C. The supernatants were moved to new tubes, and 252 µl of Exoquick (Exoquick, Systems Bioscience Inc.  cat. #EXOQ20A-1) was added to each tube. After a 1 hour incubation at 4°C samples were centrifuged at 1500 x g for 20 minutes at 4°C, and the supernatant was removed and the pellet re-centrifuged at 1500 x g for 5 minutes at 4°C to remove any excess liquid. The pellet was re-suspended by vortexing in 0.5 ml water containing 3X protease and phosphatase inhibitors with overnight agitation at 4°C. Five microliters of the suspended EVs was added to 0.5 ml PBS (1:100 dilution) and the resulting preparation of unlysed EVs was used for Nanoparticle tracking analysis (NTA). Of the remaining EV sample, 0.2 ml was diluted 1:3 with the lysis buffer M-PER (Thermo Scientific, Inc.) and designated total plasma EVs. The remaining 0.3 ml of suspended EVs received 5 µg of L1CAM antibody (biotin antihuman CD171 eBioscience cat#13-1719-82) or EpCAM antibody in 50 µl 3% BSA. Aliquots were incubated for 90 minutes at 4°C with gentle agitation. 18.75 µl Pierce™ Streptavidin Plus UltraLink™ Resin (cat. #53116 Thermo Fisher Scientific Inc,) and 21.25 µl 3% BSA were added to each sample, and gently agitated at 4°C for 30 minutes. Then, after centrifugation at 200 x g for 10 minutes at 4°C, the supernatant was removed and 50 µl 0.1 M glycine was added to each sample and the samples were vortexed. The resultant EV suspension was centrifuged at 4500 x g for 5 minutes at 4°C, and 15 µl 1M TRIS (pH 8.0) was added to adjust the pH to 7.0 and 300 µl of M-PER (Thermo Scientific, Inc.) was added.

**Nanoparticle tracking analysis:** The size and concentration of EVs were measured by NTA using NanoSight NS500 (Malvern Instruments Ltd.) per the manufacturer’s protocol. For each sample, five 20-second videos were recorded at camera level 14. Analysis was performed at detection limit 3. All samples were analyzed by a user blinded as to the source of the EV preparation (Gardiner *et al.* 2013).

**Immunoblot analysis:** Isolated EVs were diluted in M-PER (Thermo Scientific, Inc.) lysis buffer in a ratio of 1:3 supplemented with protease and phosphatase inhibitors (Roche). Lysates were vortexed for 30 seconds and sonicated for 5 minutes. Then 5 µl of the EV lysate were analyzed by SDS-PAGE followed by immunblotting with antibodies against CD9 1:500 (clone H-110, Santa Cruz Biotechnologies Inc) and ALIX 1:1000 (Cell Signaling).

**Enzyme-linked immunosorbent assays (ELISAs):** The following ELISAs were performed on samples using the manufactures’ protocols and with the following EV preparation volumes: 50 µl for mBDNF (BDNF Emax® ImmunoAssay System, Promega, cat. #G7611); 50 µl for proBDNF (Pro-BDNF [Human, Mouse, Rat] ELISA Kit, Aviscera Bioscience, cat. #s SK00752-08 and SK00752-09); 30 µl for leptinR (R&D Systems); 100 µl for IGFR (R&D Systems); 25 µl for phosphor-serine 312 IRS1 and pan-tyrosine IRS1 (MSD).

**Membrane antibody arrays:** We used the Human Obesity Antibody Array (Abcam Catalog # ab169819; Cambridge, UK) and followed the manufacturer’s protocol. Briefly, samples were sequentially incubated with paired biotinylated detector antibodies, streptavidin-horseradish peroxidase and chemiluminescent detection reagents with intervening washes in buffer. The relative amounts of immunoreaction product were quantified using Image J and each protein was normalized to the level of the positive control reference provided on each membrane. To enhance visualization of the results and facilitate comparison between time points and diet groups, we generated a color-coded heat map of the relative amounts of each protein. To generate the data depicted in Figure 1B, we isolated total EVs from plasma samples of 4 healthy subjects and proceeded with alternative immunoprecipitation for L1CAM, and EPCAM. To generate sufficient amount of protein for the arrays (~300 µg) we pooled the EV preparations. To allow direct comparison between pooled preparations of total EVs, L1CAM+ EVs, and EpCAM+ EVs, we used the same numbers of vesicles for each EV type, after measuring their concentration with NanoSight NS500 and diluting appropriately.

**Statistical analysis:** For each outcome measure (leptin receptor, pan-tyrosine to serine312 phospho-IRS1 ratio, BMI, BDNF, etc.), a separate repeated measures mixed model analysis was conducted to examine the effects of the between-subjects factor “diet type” (PR diet vs. control diet), the within-subjects factor “time point” (baseline vs. after 1 month of diet) and their interaction term; the concentration of EVs by NTA was used as a covariate to control for differential EV content in the analyzed plasma samples. Including the concentration of EVs as covariate was particularly important given their baseline difference between PR and control diet groups. An Auto-Regressive (AR1) covariance matrix and Restricted Maximum Likelihood Estimation (REML) was used. We assessed model fit using the AIC and BIC criteria (lower values indicate better fit) and determined that AR1 was in fact the best fitting model, as it is often the case for repeated measures analyses. To examine differences between the 1-month visit and baseline separately for the two diet types, and between the PR and control diets at each time point, we examined post-hoc tests for the diet type versus time point interaction term employing the Least Significant Difference (LSD) statistic. This analytic approach is common in the calorie restriction literature (Weiss *et al.* 2006). Criteria for significance were set at alpha of 0.05 for all analyses. To examine within-group changes for age, various body composition measures, lipids and PSA, we performed ANOVA within each group including the within-subjects factor “time point”. To examine between-group differences for age, various body composition measures, lipids and PSA after 1 month of diet we performed ANOVA of the follow-up values including the between-subjects factor “diet type” without and with adjusting for baseline values (Supplemental Table 1).

**Supplemental references:**

Gardiner C, Ferreira YJ, Dragovic RA, Redman CW, Sargent IL (2013). Extracellular vesicle sizing and enumeration by nanoparticle tracking analysis. *J Extracell Vesicles*. **2**.

Goetzl EJ, Boxer A, Schwartz JB, Abner EL, Petersen RC, Miller BL, Kapogiannis D (2015). Altered lysosomal proteins in neural-derived plasma exosomes in preclinical Alzheimer disease. *Neurology*. **85**, 40-47.

Kapogiannis D, Boxer A, Schwartz JB, Abner EL, Biragyn A, Masharani U, Frassetto L, Petersen RC, Miller BL, Goetzl EJ (2015). Dysfunctionally phosphorylated type 1 insulin receptor substrate in neural-derived blood exosomes of preclinical Alzheimer's disease. *FASEB J*. **29**, 589-596.

Mustapic M, Eitan E, Werner JK, Jr., Berkowitz ST, Lazaropoulos MP, Tran J, Goetzl EJ, Kapogiannis D (2017). Plasma Extracellular Vesicles Enriched for Neuronal Origin: A Potential Window into Brain Pathologic Processes. *Front Neurosci*. **11**, 278.

Weiss EP, Racette SB, Villareal DT, Fontana L, Steger-May K, Schechtman KB, Klein S, Holloszy JO (2006). Improvements in glucose tolerance and insulin action induced by increasing energy expenditure or decreasing energy intake: a randomized controlled trial. *Am J Clin Nutr*. **84**, 1033-1042.
